# Periodontal disease and atherosclerosis from the viewpoint of the relationship between community periodontal index of treatment needs and brachial-ankle pulse wave velocity

**DOI:** 10.1186/1471-2458-6-131

**Published:** 2006-05-14

**Authors:** Koichi Miyaki, Katsunori Masaki, Mariko Naito, Toru Naito, Keika Hoshi, Asako Hara, Shugo Tohyama, Takeo Nakayama

**Affiliations:** 1 Department of Preventive Medicine and Public Health, School of Medicine, Keio University, Tokyo, Japan; 2 Department of Molecular Epidemiology, Medical Research Institute, Tokyo Medical & Dental University, Tokyo, Japan; 3 Department of Preventive Medicine/Biostatistics and Medical Decision Making, Nagoya University Graduate School of Medicine, Aichi, Japan; 4 Section of General Dentistry, Department of General Dentistry, Fukuoka Dental College, Fukuoka, Japan; 5 Japan Council for Quality Health Care, Tokyo, Japan; 6 Department of Health Informatics, Kyoto University School of Public Health, Kyoto, Japan

## Abstract

**Background:**

It has been suggested that periodontal disease may be an independent risk factor for the development of atherosclerosis. However, the relationship between periodontal disease and atherosclerosis has not been fully elucidated. This study aimed to assess the effects of periodontal disease on atherosclerosis.

**Methods:**

The study design was a cross-sectional study. Subjects were 291 healthy male workers in Japan. We used the Community Periodontal Index of Treatment Needs (CPITN) score, average probing depth and gingival bleeding index (rate of bleeding gums) to assess the severity of periodontal disease. We also used the Brachial-Ankle Pulse Wave Velocity (baPWV) as the index for the development of atherosclerosis.

**Results:**

The unadjusted odds ratio (OR) of atherosclerosis in relation to the CPITN score was 1.41 [95% CI: 1.16–1.73]. However, after adjustment for age, systolic blood pressure and smoking, the CPITN score had no relationship with atherosclerosis (adjusted OR: 0.91 [0.68–1.20]).

**Conclusion:**

Our results show no relationship between mild periodontal disease and atherosclerosis after appropriate adjustments.

## Background

For many people, periodontal disease causes problems in day-to-day life due to loss of teeth. Recently, it has been argued that periodontal disease may be an independent risk factor for the development of atherosclerosis. The potential mechanisms that could explain a role for periodontal disease in atherosclerosis are general inflammatory mechanisms and specific bacterial interactions. One hypothesis is that the cause of atherosclerosis is direct injury to vascular endothelial cells by an infecting organism; another is that inflammatory cytokines contribute to atherosclerosis [1,2]. It has been reported that the infected or inflamed area in periodontitis is associated with macrophage activation via increased serum lipopolysaccharide concentrations [3]. It has also been reported that subjects with advanced periodontal disease had endothelial dysfunction and evidence of systemic inflammation, possibly placing them at an increased risk for cardiovascular disease [4]. The relationship between periodontal disease and diseases of the blood vessels, such as peripheral vascular disease and coronary heart disease (CHD), has been discussed in several reports [5-9]. For example, a meta-analysis of nine cohort studies (eight prospective and one retrospective) comparing individuals with and without periodontal disease showed that the relative risk (RR) of cardiovascular events was 1.19 [1.08–1.32]. When the outcome was restricted to stroke only, the RR was 2.85 [1.78–4.56] [9].

More than 70% of all people have gum problems [10]. Among Japanese, the prevalence of advanced periodontal disease is 32% in people in their 40 s and 47% in people in their 50 s [11]. Advanced periodontal disease is defined by a Community Periodontal Index score (CPITN score) of 3–4. The CPITN score, which is defined by the World Health Organization (WHO) protocol and provides a standardized means of measurement, makes it easy to compare reports. Periodontal disease is one of the most widespread diseases in the world [12], and if it affects atherosclerosis, it must be considered an important aspect of public health. Therefore, it is essential to clarify the relationship between periodontal disease and atherosclerosis.

We defined atherosclerosis by using Pulse wave velocity (PWV), an index of arterial stiffness [13,14] regarded as a non-invasive marker of vascular damage [15-17]. Previous studies have shown that PWV is a marker of the severity of cardiovascular disease [18] and a predictor of future cardiovascular events [19-21]; moreover, it can be applied as a screening tool for cardiovascular risk in a general population [16,22]. Recently, a simple device for measuring brachial-ankle PWV (baPWV) has been developed and made available [23,24].

In this study, we assessed the effect of periodontal disease on atherosclerosis from the viewpoint of the relationship between CPITN score and Brachial-Ankle Pulse Wave Velocity (baPWV), by which we non-invasively assessed the progress of atherosclerosis.

## Methods

### Study subjects and design

This was a cross-sectional study. The setting was a Japanese chemical company. After receiving approval from the ethical committee of Keio University School of Medicine, we obtained informed consent from 291 male employees of the chemical company who were selected for participation in the cross-sectional study. In September 2004, we gave the participants questionnaires about their daily life and dental habits. Oral and medical examinations were performed in October 2004, at which time the questionnaires were also collected.

### Periodontal measurements

We used the CPITN score, average probing depth and gingival bleeding index (rate of bleeding gums) to determine the severity of periodontal disease. Dental examinations were conducted by two dentists who, according to the WHO protocol, used flat dental mirrors and periodontal probes [25]. Teeth numbers 2, 3, 8, 14, 15, 18, 19, 24, 30 and 31 were studied, and six segments of each tooth were evaluated. Probing depth was measured at two locations per index tooth. Each sextant was designed as either healthy (Score 0), bleeding but no dental calculus detected (Score 1), calculus detected but no pockets (Score 2), a probing depth of more than 4 mm (Score 3) or a probing depth of more than 6 mm (Score 4), according to the highest score recorded at the index teeth. The highest score was recorded as the CPITN score of the participant. The assignment of the two dentists to subjects was carried out at random. Interexaminer agreement between the dentists was tested at baseline and calculated using the Kappa statistics. The same dentists carried out the dental examinations during the entire study period.

### Medical measurements

We used baPWV as the index for atherosclerosis. Nurses measured the baPWV and blood pressure of each subject twice using a baPWV/ABI device (Nippon Colin, Aichi, Japan) while the subject was at rest in a supine position. This device, approved by the US Food and Drug Administration as VP-2000/1000, can monitor bilateral brachial and ankle pressure wave forms simultaneously using the volume plethysmographic method, with two optional tonometry sensors for carotid and femoral arterial wave measurements. The nurses practiced baPWV measurements beforehand and made arrangements for standardization.

Age and smoking status were self-reported, and medical history was acquired by interview. We applied pack-years (smoking period [years] × number of packs [/day]) as the cumulative smoking index. Height, weight, systolic and diastolic blood pressures (SBP and DBP), fasting blood glucose level, serum lipid levels [total cholesterol, triglyceride and high-density lipoprotein (HDL) cholesterol] and HbA1c were measured in all subjects.

### Statistical analyses

The distributions of triglyceride, HDL cholesterol and HbA1c were skewed and regarded as log-normal. Therefore, these data were logarithmically transformed, and the Student's *t*-test was applied. For age, as the distribution was skewed and not log-normal, the Wilcoxon's rank sum test was applied. The other variables were regarded as normally distributed, and the Student's *t*-test was applied.

We divided the subjects based on CPITN score, average probing depth and gingival bleeding index. Subjects with CPITN scores of 0, ≧1, ≧3 and ≧4 were defined as healthy, having periodontal disease, having advanced periodontal disease and having severe periodontal disease, respectively. Similarly, for average probing depth and gingival bleeding index, we placed the subjects in four groups. The boundary values for average probing depth were 2 mm, 3 mm and 4 mm, and those for gingival bleeding index were 25%, 50% and 75%.

We defined atherosclerosis as baPWV ≧1400 (cm/sec), which is an independent variable for risk stratification by the Framingham score and for the discrimination of patients with atherosclerotic cardiovascular disease. It is the commonly used cut-off value in clinical practice [16].

Odds ratios (ORs) and 95% CIs were calculated using logistic regression analysis. All statistical analyses were performed using SPSS for Windows version 12.0 (Statistical Product and Service Solutions, IL, USA), and statistical significance was accepted at *p *< 0.05.

## Results

Mean age and body mass index were 46.6 ± 11.5 years and 23.4 ± 3.66 kg/m^2 ^(mean ± SD), respectively, which are typical values in healthy male Japanese workers. The maximum and minimum ages were 21 and 63. The biochemical values of blood did not reveal any significantly abnormal characteristics. The distribution of the CPITN score was typical in Japanese people and in people from other countries [10,12,26-28]. The case-control ratio was 0.99 (case: N = 145, control: N = 146). Between the case and control groups, significant differences were found in age, blood pressure, pack-years, periodontal measurements and other variables (Table 1). The periodontal conditions of the case group were worse than those of the control group. For example, in the case group, the prevalence of severe periodontal disease was about one-ninth, but in the control group, it was about one-fourth (Table 1). [Note: I don't quite understand what "one-ninth" and "one-fourth" mean in the above sentence.] The agreement between examiners was fairly high (Kappa statistics = 0.767, *p *value = 0.029).

The results of logistic regression are shown in Tables 2 and 3. The unadjusted ORs of atherosclerosis relating to the CPITN score, average probing depth and gingival bleeding index were 1.41 [1.16–1.73], 1.77 [1.35–2.32] and 2.03 [1.04–3.95], respectively (Table 2). However, after adjustment for age, no significant risk levels were found in CPITN score, average probing depth and gingival bleeding index (Ors = 0.98 [0.77–1.25], 1.09 [0.80–1.47] and 1.01 [0.46–2.19], respectively). In addition, after adjustment for age, SBP and pack-years were less than 1.0 (0.91 [0.68–1.20], 0.99 [0.70–1.38] and 0.71 [0.28–1.79], respectively). Age, SBP and pack-years were the major confounding factors for baPWV.

The results showed that mild periodontal disease tended to have a weaker relationship with atherosclerosis than did severe periodontal disease (Table 3). The adjusted OR for men with a CPITN score of 0–3 compared with men with a CPITN score of 4 was 1.13 [0.50–2.53]. Similarly, the OR for a CPITN score of 0–2 versus a CPITN score of = 3–4 was 0.58 [0.26–1.29]. The OR for men with a CPITN score of 0 versus a CPITN score of 1–4 was 0.50 [0.18–1.42]. This trend was observed not only for CPITN score but also for the average probing depth and gingival bleeding index (average probing depth: 1.34 (0–3 vs. 4), 1.02 (0–2 vs. 3–4), 0.42 (0 vs. 1–4); gingival bleeding index: 1.14, 0.81, 0.64).

## Discussion

Despite the existence of many reports, the relationship between periodontal disease and atherosclerosis has not been fully elucidated. Because the absence of a standard definition and measures for periodontal disease complicate the interpretation of results, as do potential confounding risk factors that are common to both conditions, we have no basis for comparison [29]. In the fairly recent past, a report was published quantifying and comparing the severity of periodontal disease and coronary-artery calcification [30]. However, that report referred only to the coronary artery, and calcification is a poor marker of atherosclerosis. Therefore, an assessment of the progress of periodontal disease and atherosclerosis has been necessary.

We used three indices of periodontal disease: CPITN score, average probing depth and gingival bleeding index. Average probing depth is similar to AL. Although AL accurately indicates gum recession and is the best index of periodontal disease, its measurement is very time-consuming. On the other hand, it takes just 3 minutes per person to measure the average probing depth. Our investigation showed the same trend as the report that used AL (Beck's study) [31]. The CPITN score is the global standard measurement, and the method for determining this score has already been established. Within the past 20 years, the CPITN score has become widely accepted as the method of choice for epidemiological and screening studies for periodontal disease [26-28]. The gingival bleeding index indicates inflammation in the mouth, which is the major symptom of periodontal disease. We adopted baPWV as the index for atherosclerosis. BaPWV is influenced mainly by age and systolic blood pressure. In our study, we adjusted for these confounding factors. A benefit of baPWV is that it is non-invasive and easy to measure. Therefore, CPITN score and baPWV can be the standard assessment tools of periodontal disease and atherosclerosis.

In our study, before adjustment, the indices of periodontal disease showed a relationship with atherosclerosis. The ORs of atherosclerosis related to the three indices of periodontal disease were more than 1, and their *p *values were less than 0.05, which agrees with reports and hypotheses that are currently in existence [1-9,29-34]. However, after adjustments for age, these relationships were not shown (Tables 2 and 3). The ORs of atherosclerosis relating to CPITN score, average probing depth and gingival bleeding index were 0.98 [0.77–1.25], 1.09 [0.80–1.47] and 1.01 [0.46–2.19], respectively. Therefore, due to the relationship between these three indices and baPWV, we found no association between periodontal disease and atherosclerosis. Hujoel *et al*. reported that, after adjustment for known cardiovascular risk factors, gingivitis was not associated with CHD (hazard ratio (HR): 1.05, 95% CI: 0.88–1.26) in 2000 [32], and the confirmed elimination of chronic dental infections did not lead to a decreased risk of a CHD event (RR: 1.02, 95% CI: 0.86–1.21) in 2001 [35]. They also reported that the presence of periodontitis and gingivitis did not increase CHD risk among these at-risk individuals (HR: 0.97, 95% CI: 0.72–1.31; HR: 1.09, 95%CI: 0.79–1.50, respectively) in 2002 [36]. In addition, some reports have suggested that periodontal disease is not associated with CHD [37-39]. Therefore, periodontal disease is probably not associated with atherosclerosis to any large extent, if at all.

We examined the presence of a monotonic dose-response association in three ways (Table 3). With adjustment for age, SBP and pack-year, severe periodontal disease was statistically different from non-cases, but moderate periodontal disease was not. The adjusted ORs of atherosclerosis relating to periodontal disease, advanced periodontal disease and severe periodontal disease were 0.50, 0.58 and 1.13, respectively. Dose response was observed not only in periodontal disease but also in the average probing depth and gingival bleeding index. Beck *et al*. reported a similar trend [33].

There were several limitations to our study. First, the number of subjects was relatively small, and the results of this study need to be assessed in a larger sample size. However, the sample size was small because the strength of the relationship that we observed was weaker than the relationship that we had assumed from the previous studies. Second, our cross-sectional study design lacked information on the time sequence of events and so did not permit identification of the causal relationship. To investigate this relationship, it will be necessary to follow this cohort. Finally, the problem of imperfect measurement of confounders can exist. However, it is not a serious problem because adjustments were made for the major confounders. For example, the adjustment for smoking intensity is very important. Hujoel pointed out that the studies that either did not adjust or adjusted poorly for smoking showed a positive chronic periodontitis (CP)-CHD association, while the results of studies controlling for smoking suggested that CP was either not at all or weakly associated with CHD [37]. In addition, he suggested that the lack of rigorous control for smoking history was a plausible explanation for the studies that reported significant CP-CHD associations. In our study, we evaluated an integrated smoking dose as pack-years. After the appropriate adjustment including smoking intensity, we can conclude that mild periodontal disease is not associated with atherosclerosis, which is in agreement with other studies.

## Conclusion

In conclusion, we found no relationship between mild periodontal disease and atherosclerosis after appropriate adjustments from this cross-sectional study.

## Competing interests

The author(s) declare that they have no competing interests.

## Authors' contributions

MK conceived the study, designed the protocol, enrolled participants, participated in data collection and analysis, and helped to draft the manuscript. MK participated in data collection and analysis and in drafting the manuscript. MN, TN and KH gave advice on key concepts of the study and participated in data collection. AH and ST participated in data collection. TN was responsible for the study design and organization, interpretation of the data and project oversight. All authors were involved in data interpretation and contributed to the writing of the paper.

## Pre-publication history

The pre-publication history for this paper can be accessed here:



## Figures and Tables

**Table 1:** Characteristics of study participants

	Atherosclerosis (+)** (N = 45)		Atherosclerosis (-)** (N = 146)		p Value
		
	Mean	± SD	Mean	± SD	
Age	51.9	7.9	40.7	11.9	0.000*
Weight [kg]	65.4	9.3	68.2	11.3	0.027*
BMI^† ^[kg/m^2^]	23.5	3.2	23.5	3.3	0.942
Systolic blood pressure [mmHg]	143.6	16.3	127.1	11.5	0.000*
Diastolic blood pressure [mmHg]	89.0	10.6	77.2	9.2	0.000*
Total cholesterol [mg/dl]	201.9	37.7	195.3	35.3	0.139
Triglyceride [mg/dl]	164.9	129.0	132.0	126.1	0.034*
HDL^†^cholesterol [mg/dl]	53.9	15.6	54.5	13.0	0.728
Fasting plasma glucose concentrations [mg/dl]	107.6	28.8	95.0	17.0	0.000*
HbA1c^† ^[%]	5.22	0.47	5.07	0.54	0.011*
Pack-years	16.6	17.9	10.8	13.6	0.003*
baPWV^† ^[cm/sec]	1609	185.2	1275	84.8	0.000*
CPITN score^†^	2.72	1.19	2.20	1.24	0.000*
Average probing depth [mm]	3.20	1.00	2.78	0.87	0.000*
Gingival bleeding index [%]	48.8	-	40.0	-	0.042*
Prevalence of periodontal disease***[%]	90.3	-	86.2	-	0.299
Prevalence of advanced periodontal disease***[%]	73.1	-	50.7	-	0.000*
Prevalence of severe periodontal disease***[%]	24.6	-	11.6	-	0.005*
Number of teeth	25.8	5.2	26.0	5.4	0.773

† BMI: body mass index, HDL: high-density lipoprotein, HbA1c: hemoglobin A1c, baPWV: brachial-ankle pulse wave velocity, CPITN: Community Periodontal Index of Treatment Needs

* *p *value < 0.05

**Atherosclerosis is defined as baPWV ≧ 1400 (cm/sec)

*** Periodontal disease, advanced periodontal disease and severe periodontal disease are defined by CPITN scores of ≧1, ≧3 and ≧4, respectively.

**Table 2:** Odds ratios of atherosclerosis and their 95% CI-related indices of periodontal disease (CPITN score, average probing depth and gingival bleeding index) before and after adjustment for the confounding factor of baPWV^†^

	Unadjusted	Adjusted for age	Adjusted for age and SBP^†^	Adjusted for age, SBP^† ^and pack-years
CPITN† score	1.41* [1.16–1.73]	0.98 [0.77–1.25]	0.86 [0.65–1.13]	0.91 [0.68–1.20]
Average probing depth	1.77* [1.35–2.32]	1.09 [0.80–1.47]	0.92 [0.66–1.28]	0.99 [0.70–1.38]
Gingival bleeding index	2.03* [1.04–3.95]	1.01 [0.46–2.19]	0.61 [0.25–1.50]	0.71 [0.28–1.79]

^† ^SBP: systolic blood pressure, CPITN: Community Periodontal Index of Treatment Needs, baPMV: brachial-ankle pulse wave velocity

* *p *value < 0.05

Atherosclerosis is defined as baPWV ≧1400 (cm/sec).

**Table 3:** Odds ratios of atherosclerosis and their 95% CI when categorized by indices of periodontal disease (CPITN score, average probing depth and gingival bleeding index) before and after adjustment for age, systolic blood pressure and pack-years

	Unadjusted	Adjusted for age, SBP^†^, pack-years
Periodontal disease (CPITN^† ^score ≧1)	1.51 [0.71–3.19]	0.50 [0.18–1.42]
Advanced periodontal disease (CPITN^† ^score ≧3)	2.58* [1.56–4.26]	0.58 [0.26–1.29]
Severe periodontal disease (CPITN^† ^score = 4)	2.46* [1.30–4.64]	1.13 [0.50–2.53]
Average probing depth ≧2 mm	2.06 [0.81–5.28]	0.42 [0.12–1.53]
Average probing depth ≧3 mm	2.62* [1.61–4.28]	1.02 [0.52–1.99]
Average probing depth ≧4 mm	3.19 * [1.66–6.13]	1.34 [0.59–3.04]
Gingival bleeding index ≧25%	1.34 [0.82–2.09]	0.64 [0.33–1.26]
Gingival bleeding index ≧50%	1.59 [0.99–2.55]	0.81 [0.43–1.53]
Gingival bleeding index ≧75%	1.73* [1.04–3.09]	1.14 [0.56–2.29]

† SBP: systolic blood pressure, CPITN: Community Periodontal Index of Treatment Needs

* *p *value < 0.05

Atherosclerosis is defined as baPWV ≧1400 (cm/sec).
